# Supplementary material for: V-ATPase/TORC1-mediated ATFS-1 translation directs mitochondrial UPR activation in C. elegans
Source: J Cell Biol. 2022 Oct 31;222(1):e202205045. doi: 10.1083/jcb.202205045 (PMC9623136; doi:10.1083/jcb.202205045)
Supplement: Table S2 — show the list of primers used for qRT-PCR in this study. [file JCB_202205045_TableS2.docx]

**Table S2. List of primers used for qRT-PCR in this study.**

| Gene | Forward primer (5'-3') | Reverse primer (5'-3') |
| --- | --- | --- |
| *atfs-1* | GAATAAGCCTCTATGATCCGATG | GGTTGAAGCTGGGAAAGTGA |
| *hsp-6* | AGAGCCAAGTTCGAGCAGAT | TCTTGAACAGTGGCTTGCAC |
| *hsp-60* | GGAAGCCCAAAGATCACAAA | CAGCCTCCTCATTAGCCTTG |
| *cco-1* | ACCCAGATCCACTTGAGCACGC | TGTCCGGAATCTTGCTCACACATGC |
| *clec-4* | GGGATATGGAGCGACACTGG | TCGCAAATCTTCTGGCCCTT |
| *gpd-2* | AAGGCCAACGCTCACTTGAA | GGTTGACTCCGACGACGAAC |
| *lonp-1* | CGATGATGGCCATTGTGCAG | CGCTTTGAAACATCAATTTCATCCA |
| *let-363* | TCGATGGGCTGAACAAACGA | GACACCTGCAGCTTCCTCTT |
| *rheb-1* | ACGTGATTCTTGTGAAATGGCTT | ACGAAACAGATTCGGGAAAAGA |
| *timm-23* | GTTTCCCGGCAAATGACACC | AACGATTGCTCCAGACGGTT |
| *vha-1* | TACGGTATGATCGTCGCCCT | AGCGGCGATGAAGGAATTGT |
| *raga-1* | GGATCCGGCAAGACTTCGAT | AACTCTCGGCTTTCCACGTC |
| *hsp-4* | GACATCGAGCGCATGATCAA | CCTTGTCGGCGATTTGAGTT |
| *dve-1* | CCATGCAAAACGCCAACTCA | TTTGCATCCATGTCGGGTGA |
| *xbp-1s* | CGTGCCTTTGAATCAGCAGTG | CGAGGTGTCCATCTTCTTGTT |
| *hsf-1* | CCCGGAAAATGGATTTGAT | CCGGTGAATGTGGGAAGA |
| *daf-16* | GAGACTGTTGACAGCGGAAGA | TCTGAAATCCGAAGGAAACGATG |
| *rsks-1* | CGTCGTCTCTCTGGAGCATC | TCCATGTCCACAACGCATCA |
| *pmp-3* | GTTCCCGTGTTCATCACTCAT | ACACCGTCGAGAAGCTGTAGA |
