# Supplementary figures and images for: V-ATPase/TORC1-mediated ATFS-1 translation directs mitochondrial UPR activation in C. elegans
Source: J Cell Biol. 2022 Oct 31;222(1):e202205045. doi: 10.1083/jcb.202205045 (PMC9623136; doi:10.1083/jcb.202205045)

SourceData F3

Fig. 3A

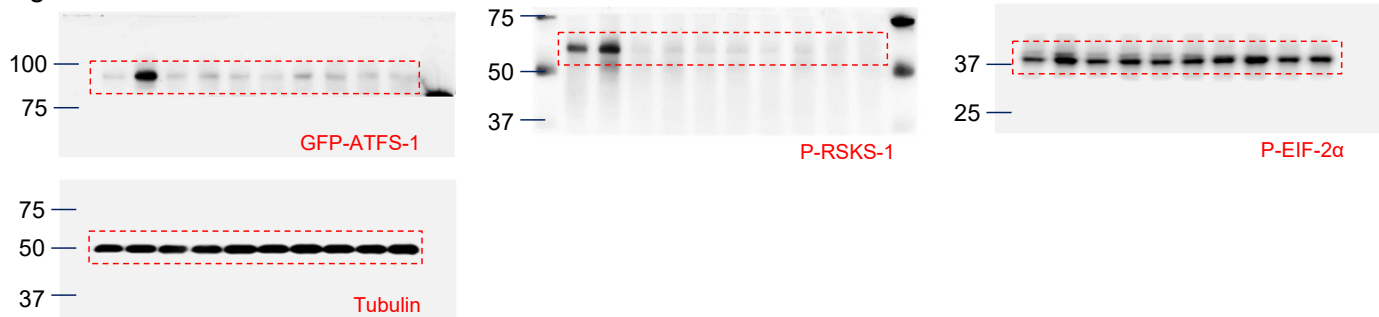

Fig. 3D

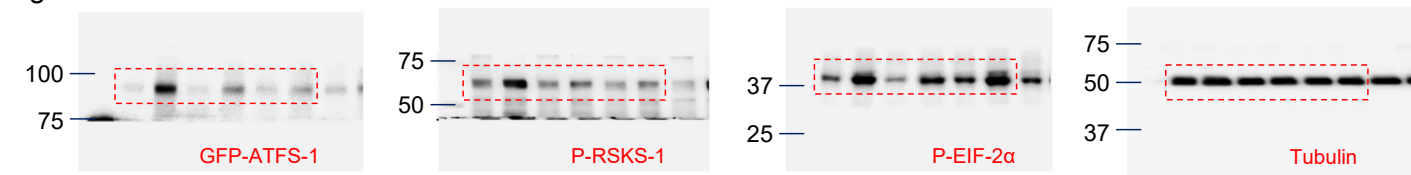

Fig. 3F

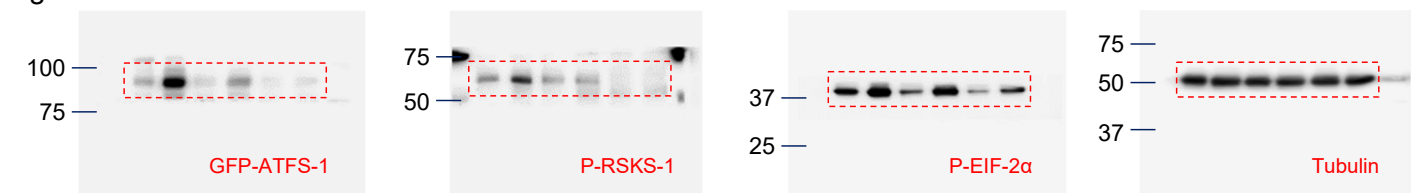

Fig. 3H

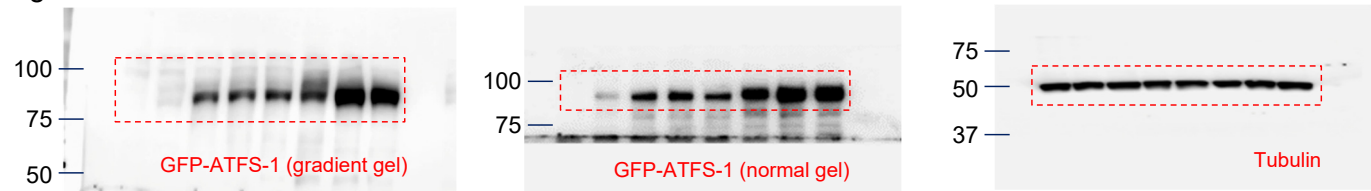

Fig. 3J

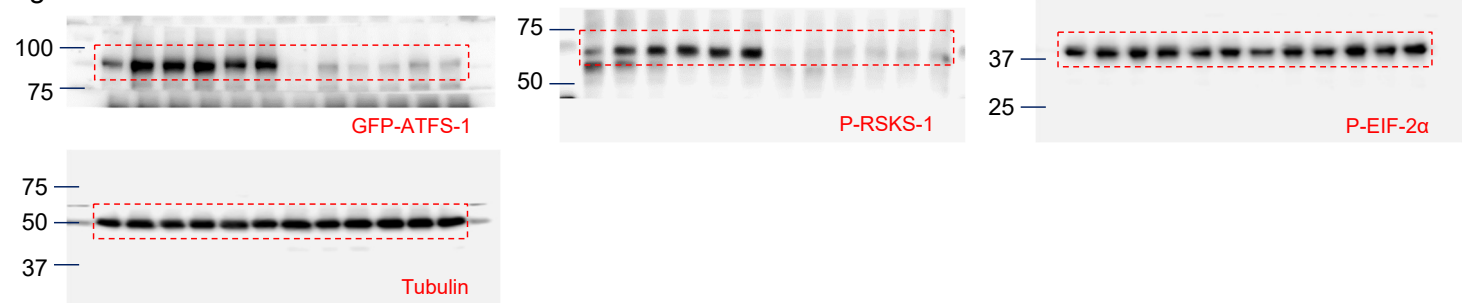

Supplement: SourceData F3 — is the source file for Fig. 3. [file JCB_202205045_SourceDataF3.pdf]

SourceData F4

Fig. 4B

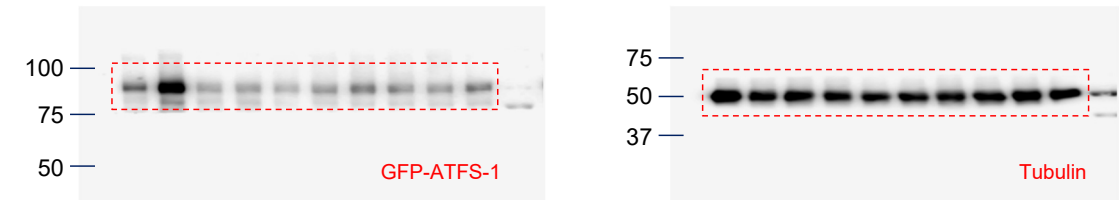

Fig. 4D

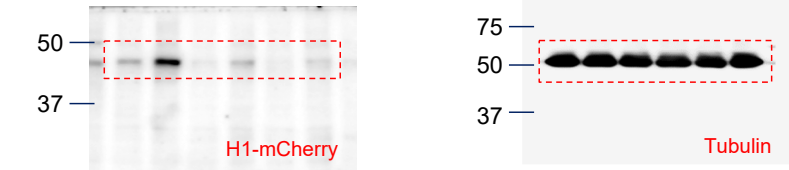

Fig. 4E

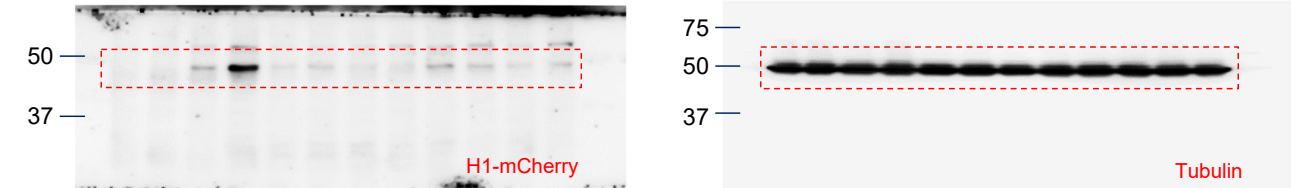

Fig. 4F

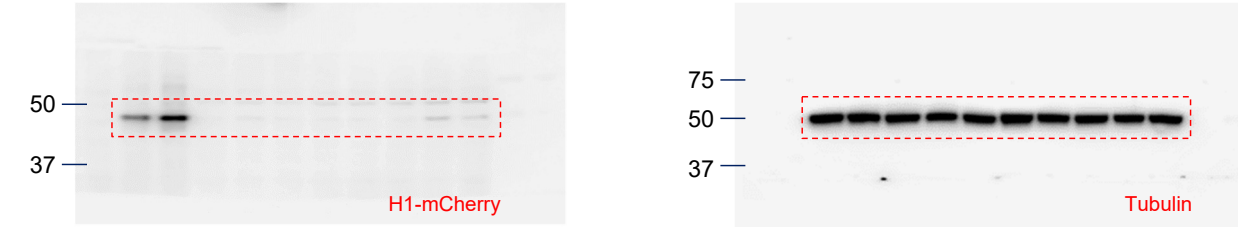

Supplement: SourceData F4 — is the source file for Fig. 4. [file JCB_202205045_SourceDataF4.pdf]

SourceData F5

Fig. 5B

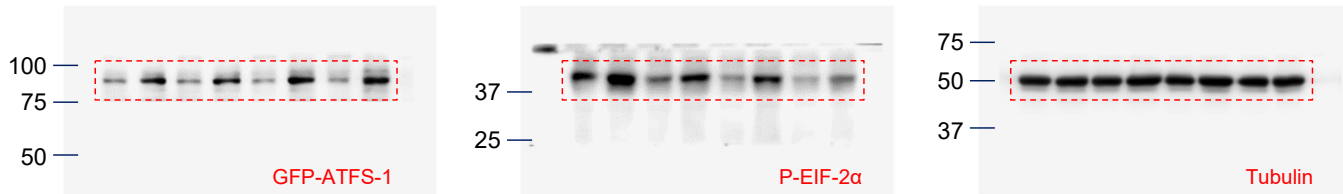

Fig. 5C

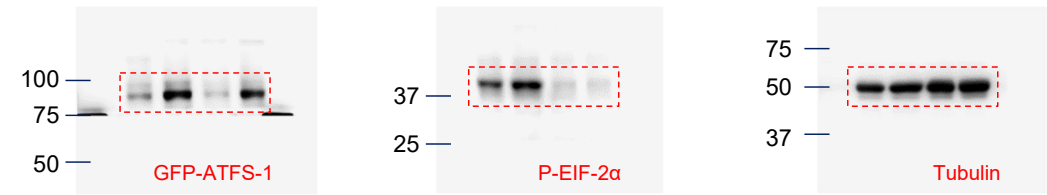

Fig. 5D

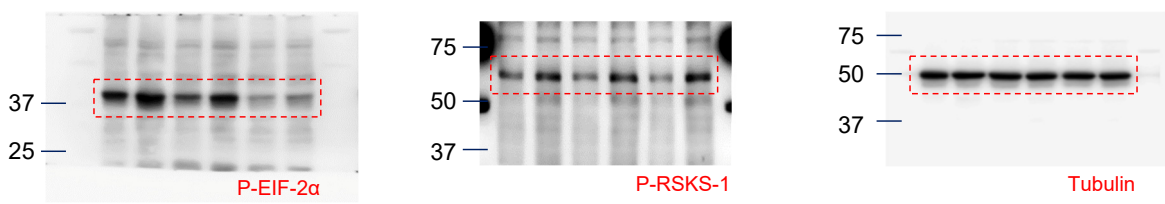

Fig. 5E

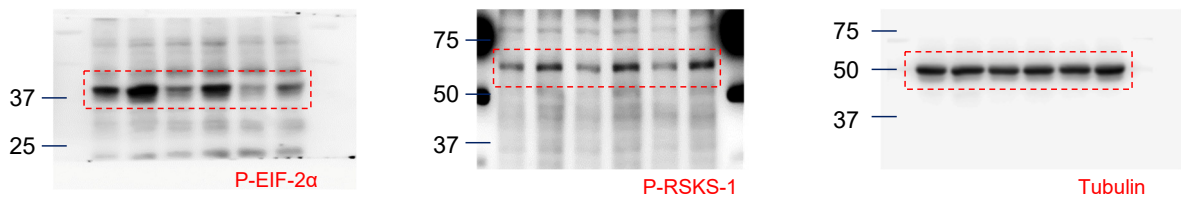

Supplement: SourceData F5 — is the source file for Fig. 5. [file JCB_202205045_SourceDataF5.pdf]

SourceData FS2

Fig. S2A

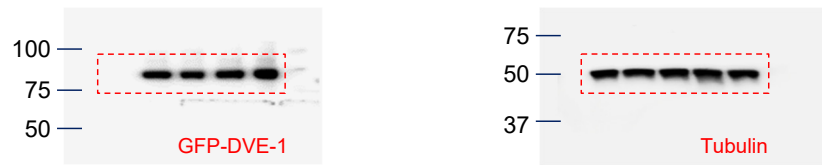

Fig. S2B

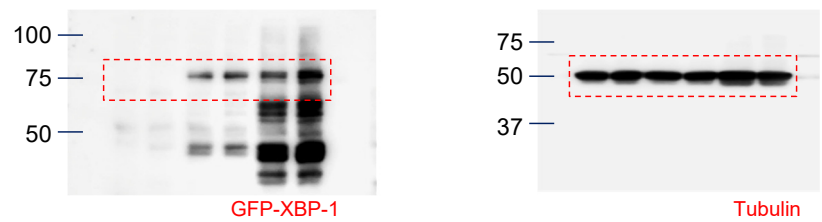

Fig. S2E

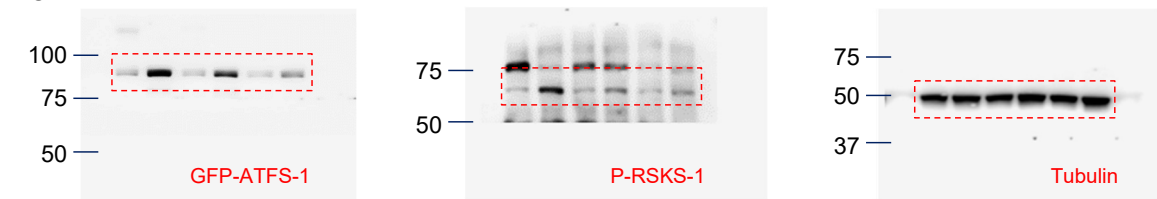

Fig. S2I

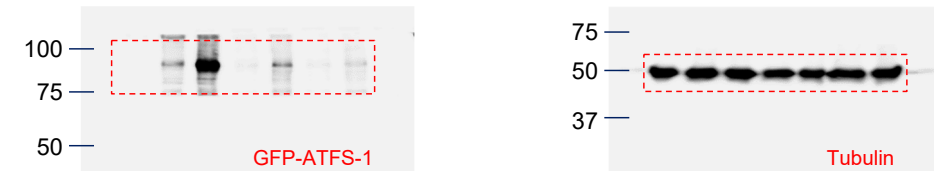

Supplement: SourceData FS2 — is the source file for Fig. S2. [file JCB_202205045_SourceDataFS2.pdf]
